# Determinants of Patient Satisfaction With Telemental Health Services in Germany: Representative Cross-Sectional Postpandemic Survey Study

**DOI:** 10.2196/65238

**Published:** 2025-05-29

**Authors:** Ariana Neumann, Hans-Helmut König, André Hajek

**Affiliations:** 1 Department of Health Economics and Health Services Research, University Medical Center Hamburg-Eppendorf, Martinistraße 52, Hamburg, 20246, Germany, 49 40 7410 54202, 49 40 7410 40261

**Keywords:** telemental health, telepsychiatry, teletherapy, telemedicine, digital health, patient satisfaction, patient preferences

## Abstract

**Background:**

Increasing patient satisfaction with telemental health services is crucial for promoting widespread implementation and ensuring consistent utilization rates in the future, where these services could be a beneficial addition to routine mental health care. Nevertheless, knowledge regarding determinants of patient satisfaction with telemental health services is very limited.

**Objective:**

This study aimed to identify determinants of patient satisfaction with telemental health services.

**Methods:**

A cross-sectional, quota-based (quotas: gender and age group), web-based survey was conducted in December 2023 in Germany. The sample included individuals aged 18‐74 years who had received telemental health services since March 2020 (N=854). Patient satisfaction with video, telephone, and asynchronous services was measured using the Telemedicine Satisfaction Questionnaire or the Client Satisfaction Questionnaire adapted to internet-based interventions. The association of socioeconomic, access, health, psychosocial, personality, and COVID-19-related factors, as well as patient preferences and provider characteristics with patient satisfaction, was tested using multiple linear regressions.

**Results:**

A positive patient attitude towards telemental health services and greater fear of COVID-19 as well as a positive and open provider attitude and higher provider skills for using the services were positively associated with patient satisfaction in all service groups (*P*<.05). Furthermore, the patients’ educational level, employment status, relationship status, certain personality factors, technology commitment, loneliness, self-efficacy, and internet connection quality at home were significantly associated with satisfaction in at least 1 service group. Physical and mental health determinants were not significantly associated with the outcome.

**Conclusions:**

Satisfaction with telemental health services is particularly associated with psychosocial characteristics, individual preferences, and attitudes of patients, which should be considered when addressing target groups for the services. Furthermore, positive provider attitudes towards and higher skills for using the services are heavily associated with patient satisfaction. Training and support for providers should be prioritized to promote patient satisfaction and widespread use of future services.

## Introduction

### Background

Mental illnesses are a leading cause of disability and were estimated to account for up to 16% of global disability-adjusted life years [1]. Even though the mental illness incidence rate is predicted to slowly decrease over the next years, the number of affected individuals and deaths due to mental illnesses is expected to grow further [2]. Particularly in the wake of the COVID-19 pandemic, an increase in the prevalence of mental illnesses was observed [3,4].

To deal with the increased demand for mental health care and the limited availability of in-person services during the pandemic, telemental health services have become indispensable. According to the National Institute of Mental Health [5], telemental health is defined as the use of telecommunications or videoconferencing technology to provide mental health care services. Overall, a 154% increase in telehealth visits in March 2020 compared to March 2019 was observed in the United States [6]. The specialty with the highest telemedicine use during the pandemic was behavioral health [7]. In a sample of mental health care providers from Florida (United States), the number of providers using telemedicine daily increased from 17% to 40% during the pandemic, and they expected to continue using telemedicine services as much or even more in the future [8]. Accordingly, the global use of telemental health services remained at a greater level compared to the prepandemic period and is anticipated to further expand in future mental health care [9].

Telemental health services can be delivered via synchronous services (eg, video or telephone calls), asynchronous services (eg, mobile apps, web-based interventions, and email), or hybrid combinations [10]. These service types have different approaches and are often used for different purposes in clinical practice [11]. Whereas the direct real-time interactions delivered via synchronous services allow almost similar treatment to in-person visits, asynchronous services are rather used for assessment or as a supplement to in-person care [11]. Telemental health services were found to be effective. Multiple reviews evaluated the effectiveness of synchronous services and suggested similar outcomes to in-person services as well as high diagnostic reliability between virtual and in-person visits [12-14]. For example, a meta-analysis of 20 randomized controlled trials by Shaker et al [14] found comparable results (synchronous video vs in-person consultations) for treatment efficiency, patient satisfaction, attrition rates, and working alliance in patients diagnosed with posttraumatic stress, mood, and anxiety disorders. Recent reviews also point towards the clinical effectiveness of asynchronous telemental health services [15-18]. For instance, a systematic review by O’Keefe et al [15] examined that asynchronous services may improve psychiatric symptoms and adherence rates, and are effective for diagnosis and scheduling activities.

General strengths of telemental health services include increased access to care, cost and time savings as well as greater convenience and flexibility (eg, [19,20]). Therefore, telemental health services have the potential to address and help mitigate future mental health care challenges such as physician shortages or increased demand for care due to population aging or in refugee populations, stigmatization associated with visiting mental health facilities, and provision of care in rural areas [20,21]. Telemental health services enable location-independent care, which can help to balance out supply shortages (eg, in rural areas and for specific or minority patient groups) and eliminate access issues (eg, for mobility-restricted individuals or long travel times). The services can be adapted to the individual preferences and needs of patients and offer some degree of anonymity [19,20].

Patients were found to be greatly satisfied with telemental health services [12-15]. A review by Sharma and Devan [12] highlighted that particularly in children and adolescents, satisfaction rates were high. They found that patients valued strengths such as greater ease in discussing sensitive topics and comfortability. In addition, specific patient groups including patients with limited mobility, patients living in rural areas as well as patients from prison or forensic settings were especially satisfied [12]. Furthermore, patients seem to prefer hybrid models for future mental health care [12,19]. High patient satisfaction is crucial for promoting widespread acceptance of telemental health services and preventing attrition in future remote mental health care. Knowledge about the determinants of patient satisfaction with these services is essential to ensure and enhance satisfaction with these services. Nevertheless, the determinants of patient satisfaction with telemental health services have been scarcely investigated thus far.

In response to this research gap, we recently published a systematic review regarding determinants of patient use and satisfaction with synchronous telemental health services during the COVID-19 pandemic [22]. Considering information from 10 studies on patient satisfaction, we mostly observed nonsignificant associations of socioeconomic factors, health, service factors, experience, psychosocial factors, and facilitating conditions with satisfaction. However, potential relationships with some psychosocial (eg, COVID-19-related stress or fear) and service factors (eg, greater satisfaction with video compared to telephone services) were identified. Furthermore, the need for future studies, particularly focusing on effort and performance expectancy, psychosocial factors as well as facilitating conditions was highlighted [22]. More recent studies also found no association of patient satisfaction with socioeconomic determinants (such as gender, age, marital status, or occupation), insurance status, psychiatric diagnosis, or frequency of telemental health service use [23-26]. However, relationships of higher education, urban residence, and low or high income with patient satisfaction were found in individual studies [23,24,26]. In addition, technical difficulties, unstable internet access, and worsening of depressive or anxiety symptoms were associated with lower patient satisfaction in one study [23]. Only limited research exists on asynchronous telemental health services. Individual studies found associations of women gender, higher self-regulation, and patient status with patient satisfaction [27,28].

As a result of the COVID-19 pandemic, synchronous as well as asynchronous telemental health services were also widely implemented in Germany, which is the focus of our study. Already in November 2019, the Digital Healthcare Act was approved to promote the digital transformation of the German health care system [29]. For instance, this includes regulations that facilitate the prescription of mobile health apps, provision of web-based video consultations, and integration of digital provider networks. Furthermore, the Digital Health Applications Ordinance [30] was approved in April 2020, which regulates policies for digital health applications in Germany regarding security, quality, data protection, and data security. Consequently, the use of digital health applications increased during the pandemic in Germany. The overall telemedicine use among contract physicians and psychotherapists increased from 6.1% (2019) to 24.6% (2021) [31]. Telemental health services were used most frequently in Germany and accounted for 86.3% of all offered telemedicine services in 2021 [31].

Future mental health care challenges are highly relevant to high-income countries also including Germany (eg, physician shortages, aging population, and mental health care for refugees) and research indicates unmet needs in the current provision of mental health care in Germany [32]. Telemental health services could play a significant role in addressing these issues. Very few studies from Germany have found high patient satisfaction with the services, which was associated with newer patient status (ie, patients who had just started outpatient telephone therapy compared to patients in ongoing therapy who switched to telephone services) and video services (compared to telephone services) but not with age, gender, symptom severity, stress, psychosocial functioning, or computer skills [28,33-35].

### Objective

Increasing patient satisfaction with telemental health services is vital for their successful and widespread implementation, potentially serving as a valuable addition to the future of routine mental health care. Currently, there is generally limited evidence regarding the determinants of patient satisfaction with telemental health services and multiple research gaps exist that are listed as follows:

1. Psychosocial determinants and individual patient preferences may be critical, as they potentially are modifiable towards greater patient satisfaction (eg, [36,37]).
2. There is unclear evidence regarding differences in socioeconomic, health, and access factors, and their impact on patient satisfaction. These factors might pose additional hurdles for telemental health service users, potentially impacting their satisfaction levels.
3. Little is known about the relationship of provider characteristics and patient satisfaction. Since provider characteristics were found to be connected to patient satisfaction with telemedicine (eg, [38,39]), they might also be a key factor contributing to patient satisfaction.
4. Studies considering asynchronous services and the postpandemic context (ie, simultaneous availability of in-person and remote services) are lacking.

Nevertheless, these factors may be critical for better understanding patient preferences in the current landscape of mental health care. Therefore, our study aimed to identify determinants of patient satisfaction with telemental health services in Germany.

## Methods

### Sample

Cross-sectional data of the general adult population in Germany (18‐74 y) were collected by the market research firm Bilendi, an International Organization for Standardization (ISO) 20252:2019-certified sample provider. The inclusion criteria were as follows: individuals who had been using mental health services since March 2020 were considered for participation in the web-based survey. In addition, participants were recruited based on quotas for gender and age, which were derived from mental health service utilization rates in Germany [32,40] to promote the representativeness of our sample. The web-based questionnaire was pretested in November 2023 including 13 individuals (n=8 women) to assess clarity and accuracy of the questionnaire and check for potential bias. Very minor amendments were made after pretesting. Eventually, the data collection was carried out between December 1 and December 15, 2023. Overall, 2178 adults took part in the web-based survey. Since we wanted to examine satisfaction among patients who already had experience with telemental health services, only individuals who reported past use of these services were included in our study (N=854).

### Ethical Considerations

Participants received compensation from Bilendi, which included real-time cash payments ranging from 0.90€ to 1.80€ (conversion rate in 2023: US $1=€0.924) upon completion of the questionnaire. After data collection was finalized, Bilendi supplied an anonymized data set. All participants provided informed consent and Bilendi panel members are able to opt out of the panel at any time. The study was approved by the Local Psychological Ethics Committee of the University Medical Center Hamburg-Eppendorf (LPEK-0683).

### Dependent Variable

Patient satisfaction with telemental health services was measured using validated instruments. For synchronous telemental health services, including video and telephone services, the Telemedicine Satisfaction Questionnaire was used (TSQ; [41]). The questionnaire consists of 14 items with a 5-point Likert scale (ranging from 1=strongly disagree to 5=strongly agree), which assesses the quality of care, similarity to in-person encounters, and the overall perception of the interaction. Therefore, the TSQ sum score can range between 14 and 70, with higher values indicating greater satisfaction. The TSQ was evaluated in the past and shows favorable psychometric properties [41]. In our sample, Cronbach α and McDonald ω for the TSQ were 0.94 for video services and 0.93 for telephone services. Since the original version of the TSQ was published in English, the questionnaire was translated into German by 2 specialized translators from the professional translation agency Tolingo (translation carried out by the first and editing by a second specialized translator; ISO 17100-certified). Guided by existing guidelines for the cross-cultural adaptation of self-report measures (eg, [42]), we further refined the translation to enhance its semantic, idiomatic, experiential, and conceptual equivalence before pretesting the questionnaire. Experts from the fields of health services research and psychology were involved in this process.

Satisfaction with asynchronous services was measured with the Client Satisfaction Questionnaire adapted to internet-based interventions (CSQ-I; [43]). The CSQ-I consists of 8 items, which measure the general satisfaction of the patients with the intervention and the help they received as well as the likelihood of recommending and reusing the services using a 4-point Likert scale (ranging from 1=does not apply to me to 4=does totally apply to me). The overall CSQ-I score can range between 8 and 32, with higher values indicating greater levels of satisfaction. The German version of the CSQ-I showed very good reliability and construct validity [43]. When measuring patient satisfaction with asynchronous telemental health services in our study, the CSQ-I showed a Cronbach α and McDonald ω of 0.95.

### Independent Variables

Multiple determinants and their association with patient satisfaction with telemental health services were observed. Based on theoretical consideration, past findings and research gaps (eg, [22]), socioeconomic, access, health, psychosocial, personality, and COVID-19-related factors, as well as patient preferences (based on the Unified Theory of Acceptance and Use of Technology; UTAUT [44]), were included as determinants. In addition, we considered provider characteristics to test their potential relationship with patient satisfaction.

Socioeconomic factors that were observed in our study included gender (men, women, diverse or intersex), age, employment status (unemployed, full-time employed, part-time employed, and other), area lived in (urban, mostly urban, and rural), living situation (living with a partner in the same household, living with a partner without a common household, widowed or partner deceased, single or divorced) and migration background (no and yes). The educational level of participants was classified based on the (International Standard Classification of Education 1997; ISCED-97; [45]). The ISCED-97 levels were aggregated into 3 groups indicating a low (ISCED levels 0‐2), medium (ISCED levels 3 and 4), or high (ISCED levels 5 and 6) educational level. Furthermore, the monthly household net income was categorized into tertiles (low, medium, and high) based on 13 given income categories (1=less than €500; 2=€500 to under €1000; 3=€1000 to under €1500; 4=€1500 to under €2000; 5=€2000 to under €2500; 6=€2500 to under €3000; 7=€3000 to under €3500; 8=€3500 to under €4000; 9=€4000 to under €4500; 10=€4500 to under €5000, 11=€5000 to under €6000; 12=€6000 to under €8000; 13=€8000 or higher; conversion rate in 2023: US $1=€0.924). This assessment of household income is similar to other large surveys such as the German Health Interview and Examination Survey for Adults (Studie zur Gesundheit Erwachsener in Deutschland [46]) or the Hamburg City Health Survey [47].

Different access factors were examined. Due to differences in regulations and complexity regarding the use of telemedicine, different insurance types (statutory health insurance and private health insurance) were included as determinants. In addition, the internet connection quality at the patients’ home was considered (fast and stable; fast, but not stable; stable, but not fast; neither fast nor stable or no internet connection at home).

Health factors included the number of psychiatric diagnoses that patients received since March 2020 and the presence of chronic physical illnesses (no and yes). Furthermore, self-rated health was measured using a 5-point Likert scale (ranging from 1=very bad to 5=very good).

Psychosocial factors that were observed are loneliness and general self-efficacy. Loneliness was measured using the 6-item De Jong Gierveld Loneliness Scale [48], which has good psychometric properties [48]. Also in our sample, the scales’ Cronbach α was 0.80 and McDonald ω was 0.79. In addition, general self-efficacy was measured with the 3-item Short Scale for Measuring General Self-efficacy Beliefs (Allgemeine Selbstwirksamkeit Kurzskala; ASKU [49]). The German version of the ASKU was found to measure self-efficacy in a reliable and valid way [49]. The scales’ Cronbach α and McDonald omega were 0.89 in our study.

Personality was assessed using the Big Five Inventory–Socio-Economic Panel (BFI-S) [50]. The BFI-S contains 15 items, which measure conscientiousness, extraversion, openness, agreeableness, and neuroticism (each with 3 items). The scale was developed as part of the Socio-Economic Panel and is based on the original 44-item Big Five Inventory [51]. The BFI-S shows mostly acceptable reliability and validity [52]. Cronbach α and McDonald ω for the 5 dimensions ranged between 0.51 (agreeableness) and 0.77 (extraversion) in our sample.

COVID-19-related factors that we controlled for are COVID-19 vaccination status (no and yes) and fear of COVID-19. The 7-item Fear of COVID-19 Scale was used to measure fear of COVID-19 [53]. The German version of the 7-item Fear of COVID-19 Scale shows good psychometric properties [54] and reached a Cronbach α and McDonald ω of 0.92 in our sample.

Patient preferences that were examined are attitude towards telemental health services and technology commitment. The attitude towards telemental health services was assessed using the Unified Theory of Acceptance and Use of Technology-Patient version (UTAUT-P; [55]), which is a 14-item self-report measure to explore patient attitudes towards telepsychotherapy in clinical practice and research. The instrument is based on the well-established framework of the UTAUT [44] and was adapted to the context of patients receiving telepsychotherapy. The UTAUT-P includes four factors, which evaluate the patients’ therapy quality expectancy, convenience, ease of use, and pressure from others. It shows adequate validity and reliability [55] and Cronbach α and McDonald ω were 0.87 in our sample. Since the UTAUT-P is only available in English, it also had to be translated into German by 2 specialized translators from Tolingo for our study (translation carried out by the first and editing by a second specialized translator; ISO 17100-certified). Again, the translation was reviewed by a group of experts before the questionnaire was pretested, as recommended in existing guidelines (eg, [42]). Furthermore, technology commitment was measured with the technology commitment (Kurzskala Technikbereitschaft) 12-item short scale [56]. The scale focuses on willingness to use technology and measures technology acceptance, technology competence, and technology control beliefs. The German version of the scale has favorable psychometric properties [56] and had a Cronbach α of 0.84 and McDonald ω of 0.80 in our sample.

To observe the relationship of provider characteristics with patient satisfaction, we included the providers’ attitude towards telemental health services and the provider skills for using the services as determinants. To assess the provider attitude towards the services, participants were asked whether they agree that their mental health provider has a positive and open attitude towards telemental health services (ranging from 1=strongly disagree to 5=strongly agree). For observing provider skills, participants were asked whether they agree that their mental health care provider has the necessary skills and competencies to use telemental health services without any problems (ranging from 1=strongly disagree to 5=strongly agree).

### Statistical Analysis

Descriptive characteristics for all included variables were computed. To test the relationship between the determinants and patient satisfaction with telemental health services, multiple linear regressions were performed. Model assumptions for linear regression were checked in advance (eg, multicollinearity, homoscedasticity, and normality). Consequently, cluster-robust standard errors were calculated for all models to adjust for heteroscedasticity. By default, listwise deletion is applied in Stata SE 18.0 (StataCorp LLC) to deal with missing data. Therefore, additional analyses using full-information maximum likelihood models were conducted to further address missing data [57]. Stata (SE, version 18.0; [58]) was used for all statistical analyses and statistical significance was considered at an alpha level of *P*<.05. Finally, Cronbach alpha and McDonald omega for all included scales were estimated using the “alpha” command and “omegacoef” [59] tool in Stata.

## Results

### Sample Characteristics

In Table 1, the descriptive characteristics of all included variables are summarized. Overall, the analytic sample consisted of 854 individuals. Regarding the gender of our sample, 39.8% (340/854) of the sample were men, 60% (512/854) women, and 0.2% (2/854) diverse or intersex. The mean age of the participants was 42.3 (SD 11.1; range 18‐73) years. Regarding telemental health service use, 56.6% (483/854) of the sample reported past use of video services, 50.1% (428/854) used telephone services, and 30% (256/854) used asynchronous services. The mean patient satisfaction score for video services was 55.8 (SD 10.8; range 17‐70), for telephone services 53.3 (SD 10.9; range 14‐70), and for asynchronous services 24.1 (SD 6; range 8‐32).

**Table 1.** Sample characteristics (N=854).

Characteristics	Values
Reported use, n (%)	
Video services	483 (56.6)
Telephone services	428 (50.1)
Asynchronous services	256 (30)
Satisfaction measures, mean (SD)	
TSQ^a^ score for video services (range 17‐70, higher values indicating greater satisfaction)	55.8 (10.8)
TSQ score for telephone services (range 14‐70, higher values indicating greater satisfaction)	53.3 (10.9)
CSQ-I^b^ score for asynchronous services (range 8‐32, higher values indication greater satisfaction)	24.1 (6)
Socioeconomic factors	
Gender, n (%)	
Men	340 (39.8)
Women	512 (60)
Diverse or intersex	2 (0.2)
Age (range 18‐73 years), mean (SD)	42.3 (11.1)
Educational level (ISCED-97^c^ classification), n (%)	
Low educational level (ISCED^d^ 0‐2)	82 (9.6)
Medium educational level (ISCED 3‐4)	389 (45.6)
High educational level (ISCED 5‐6)	383 (44.8)
Employment status, n (%)	
Unemployed	192 (22.5)
Full-time employed	416 (48.7)
Part-time employed	179 (21)
Other	67 (7.8)
Household income^e^ (€), n (%)	
Low income (0 to under 2000)	254 (30.8)
Medium income (2000 to under 3500)	287 (34.8)
High income (3500 or higher)	284 (34.4)
Area lived in, n (%)	
Urban	452 (52.9)
Mostly urban	293 (34.3)
Rural	109 (12.8)
Living situation, n (%)	
Living with partner in the same household	511 (59.8)
Living with partner without a common household	48 (5.6)
Partner deceased or widowed	17 (2)
Single or divorced	278 (32.6)
Migration background, n (%)	
No	722 (84.5)
Yes	132 (15.5)
Access factors	
Insurance, n (%)	
Statutory health insurance	799 (93.6)
Private health insurance	55 (6.4)
Quality of internet connection, n (%)	
Fast and stable	617 (72.3)
Fast, but not stable	113 (13.2)
Stable, but not fast	82 (9.6)
Neither fast nor stable or no internet connection at home	42 (4.9)
Health factors	
Number of psychiatric diagnoses (range 0‐7), mean (SD)	2.2 (1.4)
Presence of at least one chronic physical illness, n (%)	
No	475 (55.6)
Yes	379 (44.4)
Self-rated health (range 1‐5), mean (SD)	3.0 (0.9)
Psychosocial factors, mean (SD)	
Loneliness (mean score range 1‐4, higher values indicate greater levels of loneliness)	2.5 (0.6)
Self-efficacy (mean score range 1‐5, higher values indicate greater levels of self-efficacy)	3.4 (0.9)
Personality, mean (SD), range 3-21	
Conscientiousness	15.7 (3.3)
Extraversion	12.6 (3.9)
Agreeableness	15.2 (3)
Openness	14.7 (3.8)
Neuroticism	14.9 (3.6)
COVID-19-related factors	
Received COVID-19 vaccination, n (%)	
No	96 (11.2)
Yes	758 (88.8)
Fear of COVID-19 (range 7‐35), mean (SD)	14.3 (7.2)
Patient preferences, mean (SD)	
Attitude towards telemental health services (range 20‐70)	47.8 (9.6)
Technology commitment (range 20‐60)	42.6 (7.8)
Provider characteristics (range 1‐5), mean (SD)	
Provider attitude towards telemental health services	3.8 (1)
Provider skills for using telemental health service use	4 (1)

a TSQ: Telemedicine Satisfaction Questionnaire.

b CSQ-I: Client Satisfaction Questionnaire adapted to internet-based interventions.

c ISCED-97: International Standard Classification of Education 1997.

d ISCED: International Standard Classification of Education.

e In 2023, the conversion rate was US $1=€0.924.

### Regression Analyses

**Figure 1.**
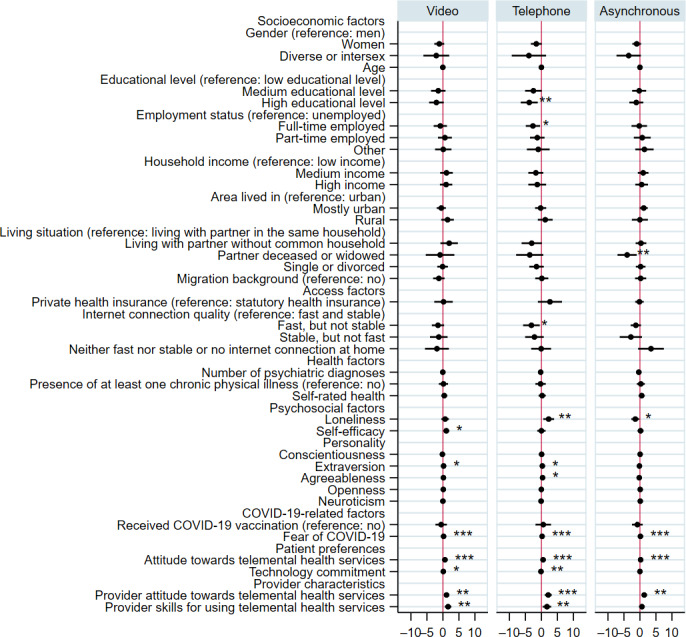
Results of multiple linear regression for determinants of patient satisfaction with video, telephone, and asynchronous telemental health services. Beta coefficients with 95% CIs are presented. ref=reference category. *** *P*<.001, ** *P*<.01, * *P*<.05.

Results of the multiple linear regression for all models are presented in Figure 1 (see Tables S1-S3 in Multimedia Appendix 1 for detailed results). Due to the varying number of users across the different service types, the sample sizes for the regression models differed. Regarding video services (n=483), we observed a significant positive association of higher self-efficacy (β=1.05, *P*=.03), extraversion (β=0.21, *P*=.03), and fear of COVID-19 (β=0.20, *P*<.001), a positive attitude towards telemental health services (β=0.63, *P*<.001), and greater technology commitment (β=0.12, *P*<.001) with patient satisfaction. A positive provider attitude towards the services (β=1.12, *P*=.004) and higher provider skills for using the services (β=1.61, *P*=.002) were additionally associated with greater patient satisfaction.

For telephone services (n=428), a significant negative association of a high educational level (β=−3.82, *P*=.005), full-time employment (β=−2.66, *P*=.02), unstable internet connection (β=−3.14, *P*=.02), and higher technology commitment (β=−0.14, *P*=.008) with patient satisfaction was found. Furthermore, higher loneliness (β=2.26, *P*=.009), extraversion (β=0.28, *P*=.03), agreeableness (β=0.35, *P*=.02), and fear of COVID-19 (β=0.21, *P*<.001), and a positive attitude towards telemental health services (β=0.52, *P*<.001) were associated with greater patient satisfaction with telephone services. In addition, a positive provider attitude towards (β=2.13, *P*<.001) and higher provider skills for using the services (β=1.73, *P*=.01) were positively associated with the outcome.

Patient satisfaction with asynchronous services (n=256) was negatively associated with having lost a partner or being widowed (β=−4.02, *P*=.009) and higher loneliness (β=−1.46, *P*=.02) in our sample. Furthermore, greater fear of COVID-19 (β=0.15, *P*<.001) and a positive attitude towards telemental health services (β=0.22, *P*<.001) as well as a positive provider attitude towards telemental health services (β=1.33, *P*=.002) were associated with higher patient satisfaction.

In the additional full-information maximum likelihood models (rather than listwise deletion), similar associations were found (see Tables S1-S3 in Multimedia Appendix 1 for detailed results). However, a few additional associations that reached statistical significance in the full-information maximum likelihood models were observed. This applies to the negative association between a medium educational level and patient satisfaction with telephone services (β=−2.99, *P*=.02). Furthermore, patient satisfaction with asynchronous services was additionally negatively associated with diverse or intersex gender (β=−3.44, *P*=.05), and agreeableness (β=−0.21, *P*=.04) and positively associated with living in mostly urban areas (β=1.23, *P*=.03), in the full-information maximum likelihood model.

## Discussion

### Principal Findings

This study aimed to explore determinants of patient satisfaction with different types of telemental health services. Examining web-based survey data of the general adult population in Germany, we identified significant relationships with patient satisfaction for some of the included determinants. Variations in associations between the different service types were observed. However, psychosocial patient characteristics (including personality and fear of COVID-19), patient preferences, and provider characteristics, were associated with patient satisfaction in all service groups. Current research regarding determinants of patient satisfaction is mostly limited to synchronous telemental health services and a small variety of determinants. Therefore, our study markedly adds to the current knowledge on a range of factors, including socioeconomic, access, health, psychosocial, personality, and COVID-19-related determinants, as well as patient preferences and provider characteristics. Furthermore, valuable insights into satisfaction with asynchronous telemental health services were gained.

### Relation to Previous Research

The majority of the included socioeconomic determinants was not associated with patient satisfaction with telemental health services, which was also observed in previous international studies (eg, [22,25]). In contrast to studies observing differences in access and additional barriers to using telemental health services for patients with low socioeconomic status [19,20,22], our findings may imply that patients are satisfied with telemental health services regardless of their socioeconomic status. Nevertheless, we observed significant negative associations of high education and full-time employment with patient satisfaction with telephone services. Highly educated and full-time employed individuals are likely to have increased access to and experience with (digital health) technologies [60]. Consequently, they may have higher expectations and are less satisfied with less technologically advanced telephone services. For asynchronous services, individuals who had experienced the loss of a partner reported lower satisfaction levels. This patient group may be especially burdened and require more extensive support. Synchronous services might be more suited for this group as they offer direct human interaction and increased social connectedness [11,17].

Regarding access factors, the patient’s health insurance type was not associated with patient satisfaction in any service group. Therefore, variations in insurance regulations (such as ease of access, complexity of the process, or usage limitations) do not seem to constitute additional barriers for telemental health service users in Germany. Even though differences in access factors (eg, digital device ownership and availability of high-speed internet) are larger in the United States compared to Germany [61], the health insurance type was also not associated with telemedicine satisfaction or use in studies with US samples (eg, [26,62]). Furthermore, the internet connection quality at home was not associated with satisfaction in almost all cases. The majority of our sample (617/854, 72.3%) had a fast and stable internet connection and only 4.9% (42/854) stated that they had slow and unstable, or no internet access at home. Since the majority of German telemental health service users seem to have a good internet connection at home, this factor does not appear to be a crucial determinant of patient satisfaction. Nevertheless, disparities in access persist and vary across different regions and countries (eg, [61,63]), which should be considered in the international context. Having an unstable internet connection at home was associated with lower satisfaction rates for telephone services. This might be unexpected since the internet is not needed for telephone calls. Patients might have been forced to switch to telephone services due to their unstable internet connection, although they actually preferred other service options (ie, video services that require a stable internet connection). However, future research is required to test these potential explanations.

None of the included health determinants were associated with patient satisfaction in the different service groups. Neither physical health factors nor comorbidity of psychiatric diagnoses were associated with patient satisfaction with telemental health services, which is in accordance with findings from studies with international samples [22]. This may suggest, for clinical practice, that telemental health services should not be restricted to certain patient groups. Furthermore, they have the potential to enhance treatment and access for specific patient populations, such as patients with limited mobility or patients experiencing social anxiety (eg, [20]).

For psychosocial determinants, associations with patient satisfaction were observed. Loneliness was positively associated with satisfaction with telephone services, but negatively associated with satisfaction with asynchronous services. This could mean that patients experiencing higher levels of loneliness prefer the direct real-time interaction provided by synchronous telephone services to satisfy their social needs. Compared to synchronous services, asynchronous services may be less personal (eg, no verbal or nonverbal cues from the provider) [11,17], which might have caused lower satisfaction levels in patients with higher loneliness. In addition, self-efficacy was positively associated with satisfaction with video services. Accordingly, self-efficacy was found to be associated with major UTAUT constructs as well as the intention to adopt and satisfaction with telemedicine services in the past (eg, [64]).

Furthermore, certain personality characteristics were associated with patient satisfaction. Higher levels of extraversion were associated with greater patient satisfaction with synchronous services. Highly extroverted individuals may feel more confident engaging in direct social interactions provided by synchronous services. In contrast, Cieślik et al [65] observed a negative association between extraversion and satisfaction with telemedicine-delivered inflammatory bowel disease care. More studies are needed to clarify this relationship. In addition, agreeableness was positively associated with satisfaction with telephone services in our study. Highly agreeable individuals may be more likely to adapt to and accept new treatment formats, such as telemedicine. In regard to previous research, agreeableness was positively associated with patient satisfaction with telemedicine services in one previous study [65] and negatively associated in another [66]. Additional significant associations with conscientiousness and openness were observed in the previous studies [65,66], which we did not find in our sample. These mixed findings highlight the need for further studies examining the relationship of personality and patient satisfaction with telemedicine services.

The fear of COVID-19 was associated with patient satisfaction in all patient groups in our study. Multiple previous studies also observed an association of COVID-19-related fear with patient satisfaction with telemedicine services [67-69]. Individuals with greater fear of COVID-19 might be particularly satisfied with telemedicine service options since they pose a lower risk of infection compared to in-person visits (eg, no physical contact while traveling to, waiting for, or during the appointment). In contrast, the COVID-19 vaccination status was not associated with patient satisfaction with telemental health services. Even though previous studies found that COVID-19 vaccination acceptance was positively associated with factors such as health literacy and trust in the health system (eg, [70,71]), it does not seem to be of significance for patient satisfaction with telemental health services.

Our findings suggest that telemental health service users hold predominantly positive attitudes towards the services. A significant relationship between the attitude towards telemental health services and patient satisfaction was found in all service groups in this study. The patient attitude towards telemental health services was evaluated using UTAUT dimensions, which have been associated with patient satisfaction with telemedicine services in past studies (eg, [72,73]). Performance expectancy, defined as the degree to which an individual believes that using the services will be beneficial [44], appears to particularly impact patient satisfaction with telemedicine services [72,73]. This expectancy may be influenced by external factors, such as the attitude of and support from providers. In addition, fostering positive initial experiences with the services (eg, [74]) and reducing technology anxiety (eg, [75]) might help to facilitate positive patient attitudes. Strengthening a positive attitude in patients may be beneficial for the successful implementation of future telemental health services. Furthermore, the patient’s level of technology commitment was positively associated with satisfaction with video services, but negatively associated with satisfaction with telephone services. Highly technology-committed patients may prefer more technologically advanced services and value the greater possibilities, which video services offer compared to simple telephone calls. This preference should be considered when choosing a synchronous service option for mental health patients. Future research on the association of patient attitude towards telemental health services and technology commitment with patient satisfaction is needed to further explore the initial observations highlighted by our study.

Patients rated provider attitudes and skills positively in our study. The provider characteristics seem to be heavily associated with patient satisfaction. A positive and open provider attitude towards telemental health services was positively associated with greater patient satisfaction in all telemental health service groups. This association has rarely been studied in previous studies in telemedicine research and seems to be crucial to promoting patient satisfaction [38,39]. A systematic review by Connolly et al [76] found that mental health care professionals hold predominantly positive attitudes towards telemental health services. However, mental health care professionals report barriers and concerns regarding the services, including technological difficulties, increased workload, perception of impersonality, safety concerns, and lack of support [76-79]. All of the UTAUT dimensions were identified as determinants of provider attitude, particularly performance expectancy [76,77,80]. Previous experience with the services and training appears to play a crucial role in fostering provider acceptance [76,78,80]. Creating offers of and improving support, guidelines, and training for telemental health care providers is essential [12,79,81], not only to decrease provider reluctance but also to increase patient satisfaction with the services. Correspondingly, better provider skills in using the services were associated with increased patient satisfaction with synchronous services. Compared to asynchronous services, providers are more directly involved in the delivery of synchronous services, and their skills seem to be especially important for patient satisfaction in this service group.

### Strengths and Limitations

In our study, we considered patient satisfaction with both synchronous and asynchronous telemental health services and tested associations with a variety of determinants. Our study adds valuable knowledge, particularly in the area of asynchronous services and for psychosocial determinants. Furthermore, the included variables were measured using validated instruments, which contributes to the credibility of our findings. In terms of gender and age, our sample was representative of mental health patients in Germany [32,40], which enabled us to gain insight into a broad spectrum of telemental health service users. The data were assessed during the postpandemic period, suggesting that our study could help to gain a deeper understanding of satisfaction with telemental health services as in-person visit availability resumed.

Nevertheless, some limitations should be noted. The cross-sectional design of our study has certain limits, which makes it difficult to draw implications regarding causality or longitudinal stability of the observed relationships. Therefore, future longitudinal and qualitative studies are needed to explore the relationships in further detail. Furthermore, we cannot completely rule out selection bias for our sample. It might be the case that some patient groups were more likely to participate in the web-based survey or to have experience with telemental health services (eg, patients with mild psychiatric symptoms, who are highly technology-committed). However, we recruited a large quota-based sample and the vast majority of mental health patients in Germany have reasonably good access to telemental health services. Finally, disparities in access, national regulations and practices regarding telemedicine vary across countries; therefore, future studies from other countries are recommended.

### Conclusion

Since determinants of patient satisfaction have been scarcely investigated thus far, we examined various determinants and their associations with patient satisfaction with telemental health services in a representative sample of mental health patients in Germany. We found that particularly psychosocial determinants, patient preferences, and provider characteristics were associated with patient satisfaction. Despite limited investigation in existing research, patient attitudes towards the services and technology commitment constitute key determinants of patient satisfaction. In addition, psychosocial determinants such as personality, loneliness, and fear of COVID-19 also appeared to have a noteworthy relationship with patient satisfaction. Therefore, when treating patients via telemental health services, it is of great importance to consider each patient individually and take their personal preferences into account.

In previous studies, clinicians were repeatedly identified as gatekeepers to telemedicine implementations [12,79], and according to our results, they also seem to play a crucial role in increasing patient satisfaction with telemental health services. Thus, finding ways to support providers and reduce barriers and misconceptions related to the provision of telemental health services should be prioritized by health care managers and researchers. In conclusion, psychosocial characteristics and individual patient preferences as well as provider characteristics may contribute to increased patient satisfaction with telemental health services, which is a key factor for the successful and widespread implementation of the services in the future.

## Supplementary material

10.2196/65238 Multimedia Appendix 1 Results of multiple linear regressions and full-information maximum likelihood models for determinants of patient satisfaction with telemental health services.

## Abbreviations

ASKU: Allgemeine Selbstwirksamkeit Kurzskala
BFI-S: Big Five Inventory–Socio-Economic Panel
CSQ-I: Client Satisfaction Questionnaire adapted to internet-based interventions
ISCED: International Standard Classification of Education
ISCED-97: International Standard Classification of Education 1997
ISO: International Organization for Standardization
TSQ: Telemedicine Satisfaction Questionnaire
UTAUT: Unified Theory of Acceptance and Use of Technology
