# Supplementary material for: Determinants of Patient Satisfaction With Telemental Health Services in Germany: Representative Cross-Sectional Postpandemic Survey Study
Source: JMIR Ment Health. 2025 May 29;12:e65238. doi: 10.2196/65238 (PMC12140380; doi:10.2196/65238)
Supplement: Multimedia Appendix 1 [file mental-v12-e65238-s001.docx]

**Multimedia Appendix 1**

**Table S1. Results of multiple linear regression and full-information maximum likelihood model for determinants of patient satisfaction with video services.**

| **Variables** | **Multiple Linear Regression** | **Full-Information Likelihood Model** |
| --- | --- | --- |
| **Socioeconomic factors** |  |  |
| Gender (ref: men) |  |  |
| Women | -1.14 | -1.18 |
|  | (0.79) | (0.75) |
| Diverse or intersex | -2.08 | -2.19 |
|  | (2.05) | (1.90) |
| Age | -0.03 | -0.03 |
|  | (0.04) | (0.03) |
| *Educational level (ref: low education)* |  |  |
| Medium educational level | -1.46 | -1.18 |
|  | (1.15) | (1.10) |
| High educational level | -2.03+ | -1.76 |
|  | (1.16) | (1.11) |
| *Employment status (ref: unemployed)* |  |  |
| Full-time employed | -0.83 | -1.05 |
|  | (1.06) | (1.00) |
| Part-time employed | 0.62 | 0.35 |
|  | (1.10) | (1.02) |
| Other | 0.10 | -0.14 |
|  | (1.30) | (1.22) |
| *Household income (ref: low income)* |  |  |
| Medium income | 1.11 | 1.11 |
|  | (1.01) | (0.96) |
| High income | 0.99 | 0.99 |
|  | (0.98) | (0.94) |
| *Area lived in (ref: urban)* |  |  |
| Mostly urban | -0.51 | -0.49 |
|  | (0.72) | (0.68) |
| Rural | 1.44 | 1.47 |
|  | (1.00) | (0.95) |
| *Living situation (ref: living with partner in the same household)* |  |  |
| Living with partner without a common household | 1.95 | 2.10 |
|  | (1.39) | (1.34) |
| Partner deceased or widowed | -0.90 | -0.88 |
|  | (2.28) | (2.11) |
| Single or divorced | -0.10 | 0.04 |
|  | (0.84) | (0.78) |
| Migration background (ref: no) | -1.27 | -1.27 |
|  | (0.91) | (0.86) |
| **Access factors** |  |  |
| Private health insurance (ref: statutory health insurance) | 0.19 | -0.14 |
|  | (1.47) | (1.34) |
| *Internet connection quality (ref: fast and stable)* |  |  |
| Fast, but not stable | -1.55 | -1.54+ |
|  | (0.98) | (0.91) |
| Stable, but not fast | -1.29 | -1.29 |
|  | (1.39) | (1.34) |
| Neither fast nor stable or no internet connection at home | -1.87 | -1.87 |
|  | (1.90) | (1.67) |
| **Health factors** |  |  |
| Number of psychiatric diagnoses | -0.03 | -0.04 |
|  | (0.25) | (0.23) |
| Presence of at least one chronic physical illness (ref: no) | 0.16 | 0.28 |
|  | (0.73) | (0.68) |
| Self-rated health | 0.42 | 0.42 |
|  | (0.48) | (0.44) |
| **Psychosocial factors** |  |  |
| Loneliness | 0.69 | 0.70 |
|  | (0.61) | (0.56) |
| Self-efficacy | 1.05* | 1.02* |
|  | (0.48) | (0.46) |
| **Personality** |  |  |
| Conscientiousness | -0.16 | -0.16 |
|  | (0.12) | (0.11) |
| Extraversion | 0.21* | 0.21* |
|  | (0.09) | (0.09) |
| Agreeableness | 0.15 | 0.16 |
|  | (0.13) | (0.13) |
| Openness | 0.11 | 0.12 |
|  | (0.11) | (0.10) |
| Neuroticism | -0.01 | -0.00 |
|  | (0.11) | (0.11) |
| **COVID-19-related factors** |  |  |
| Received COVID-19 vaccination (ref: no) | -0.56 | -0.60 |
|  | (0.91) | (0.88) |
| Fear of COVID-19 | 0.20*** | 0.22*** |
|  | (0.05) | (0.05) |
| **Patient preferences** |  |  |
| Attitude towards telemental health services | 0.63*** | 0.63*** |
|  | (0.05) | (0.04) |
| Technology commitment | 0.12* | 0.11* |
|  | (0.06) | (0.06) |
| **Provider characteristics** |  |  |
| Provider attitude towards telemental health services | 1.12** | 1.16** |
|  | (0.39) | (0.37) |
| Provider skills for using telemental health services | 1.61** | 1.51** |
|  | (0.52) | (0.49) |
| Constant | -0.62 | -0.92 |
|  | (5.05) | (4.71) |
|  |  |  |
| **Observations** | 483 | 502 |
| **R-squared** | 0.63 | 0.63 |

*Notes.* Beta coefficients are reported. Robust standard errors in parentheses. ref = reference category. *** P<.001, ** P<.01, * P<.05, +P<.10.

**Table S2.** **Results of multiple linear regression and full-information maximum likelihood model for determinants of patient satisfaction with telephone services.**

| **Variables** | **Multiple Linear Regression** | **Full-Information Likelihood Model** | |
| --- | --- | --- | --- |
| **Socioeconomic factors** |  |  |  |
| Gender (ref: men) |  |  |  |
| Women | -1.61+ | -1.37+ |  |
|  | (0.86) | (0.82) |  |
| Diverse or intersex | -3.92 | -3.67 |  |
|  | (2.73) | (2.63) |  |
| Age | -0.01 | -0.02 |  |
|  | (0.04) | (0.04) |  |
| *Educational level (ref: low education)* |  |  |  |
| Medium educational level | -2.51+ | -2.99* |  |
|  | (1.32) | (1.24) |  |
| High educational level | -3.82** | -4.22** |  |
|  | (1.35) | (1.27) |  |
| *Employment status (ref: unemployed)* |  |  |  |
| Full-time employed | -2.66* | -2.86** |  |
|  | (1.13) | (1.05) |  |
| Part-time employed | -1.30 | -1.58 |  |
|  | (1.16) | (1.09) |  |
| Other | -1.00 | -1.60 |  |
|  | (1.81) | (1.67) |  |
| *Household income (ref: low income)* |  |  |  |
| Medium income | -1.76 | -1.75 |  |
|  | (1.19) | (1.13) |  |
| High income | -1.32 | -1.31 |  |
|  | (1.41) | (1.35) |  |
| *Area lived in (ref: urban)* |  |  |  |
| Mostly urban | -0.25 | -0.17 |  |
|  | (0.89) | (0.83) |  |
| Rural | 1.18 | 1.12 |  |
|  | (1.18) | (1.14) |  |
| *Living situation (ref: living with partner in the same household)* |  |  |  |
| Living with partner without a common household | -3.04+ | -2.93+ |  |
|  | (1.61) | (1.55) |  |
| Partner deceased or widowed | -3.66+ | -3.24 |  |
|  | (2.16) | (2.07) |  |
| Single or divorced | -1.53 | -1.60 |  |
|  | (1.15) | (1.09) |  |
| Migration background (ref: no) | 0.11 | 0.22 |  |
|  | (1.04) | (0.99) |  |
| **Access factors** |  |  |  |
| Private health insurance (ref: statutory health insurance) | 2.66 | 2.88 |  |
|  | (1.92) | (1.82) |  |
| *Internet connection quality (ref: fast and stable)* |  |  |  |
| Fast, but not stable | -3.14* | -3.53** |  |
|  | (1.32) | (1.23) |  |
| Stable, but not fast | -2.18 | -2.13 |  |
|  | (1.48) | (1.41) |  |
| Neither fast nor stable or no internet connection at home | -0.10 | -0.23 |  |
|  | (1.58) | (1.42) |  |
| **Health factors** |  |  |  |
| Number of psychiatric diagnoses | -0.24 | -0.28 |  |
|  | (0.34) | (0.32) |  |
| Presence of at least one chronic physical illness (ref: no) | -0.27 | -0.15 |  |
|  | (0.82) | (0.78) |  |
| Self-rated health | 0.23 | 0.37 |  |
|  | (0.57) | (0.54) |  |
| **Psychosocial factors** |  |  |  |
| Loneliness | 2.26** | 2.16** |  |
|  | (0.86) | (0.82) |  |
| Self-efficacy | -0.01 | 0.05 |  |
|  | (0.66) | (0.62) |  |
| **Personality** |  |  |  |
| Conscientiousness | 0.06 | 0.12 |  |
|  | (0.15) | (0.14) |  |
| Extraversion | 0.28* | 0.27* |  |
|  | (0.13) | (0.12) |  |
| Agreeableness | 0.35* | 0.30* |  |
|  | (0.14) | (0.14) |  |
| Openness | -0.03 | -0.05 |  |
|  | (0.13) | (0.12) |  |
| Neuroticism | -0.11 | -0.10 |  |
|  | (0.15) | (0.14) |  |
| **COVID-19-related factors** |  |  |  |
| Received COVID-19 vaccination (ref: no) | 0.56 | 0.75 |  |
|  | (1.23) | (1.18) |  |
| Fear of COVID-19 | 0.21*** | 0.20*** |  |
|  | (0.06) | (0.05) |  |
| **Patient preferences** |  |  |  |
| Attitude towards telemental health services | 0.52*** | 0.52*** |  |
|  | (0.06) | (0.05) |  |
| Technology commitment | -0.14** | -0.13** |  |
|  | (0.05) | (0.05) |  |
| **Provider characteristics** |  |  |  |
| Provider attitude towards telemental health services | 2.13*** | 1.96*** |  |
|  | (0.56) | (0.53) |  |
| Provider skills for using telemental health services | 1.73** | 1.82** |  |
|  | (0.67) | (0.62) |  |
| Constant | 10.85+ | 10.66+ |  |
|  | (6.46) | (6.12) |  |
|  |  |  |  |
| **Observations** | 428 | 440 |  |
| **R-squared** | 0.52 | 0.52 |  |

*Notes.* Beta coefficients are reported. Robust standard errors in parentheses. ref = reference category. *** P<.001, ** P<.01, * P<.05, +P<.10.

**Table S3. Results of multiple linear regression and full-information maximum likelihood model for determinants of patient satisfaction with asynchronous services.**

| **Variables** | **Multiple Linear Regression** | **Full-Information Likelihood Model** |
| --- | --- | --- |
| **Socioeconomic factors** |  |  |
| Gender (ref: men) |  |  |
| Women | -1.01 | -1.13+ |
|  | (0.69) | (0.63) |
| Diverse or intersex | -3.52+ | -3.44* |
|  | (1.93) | (1.74) |
| Age | -0.01 | -0.01 |
|  | (0.03) | (0.03) |
| *Educational level (ref: low education)* |  |  |
| Medium educational level | -0.23 | -0.36 |
|  | (1.11) | (1.01) |
| High educational level | -1.14 | -1.00 |
|  | (1.11) | (1.00) |
| *Employment status (ref: unemployed)* |  |  |
| Full-time employed | -0.22 | -0.32 |
|  | (1.24) | (1.05) |
| Part-time employed | 0.72 | 0.44 |
|  | (1.34) | (1.14) |
| Other | 1.41 | 1.19 |
|  | (1.45) | (1.19) |
| *Household income (ref: low income)* |  |  |
| Medium income | 1.00 | 1.00 |
|  | (0.85) | (0.79) |
| High income | 0.53 | 0.53 |
|  | (1.01) | (0.94) |
| *Area lived in (ref: urban)* |  |  |
| Mostly urban | 1.18+ | 1.23* |
|  | (0.64) | (0.58) |
| Rural | -0.03 | -0.09 |
|  | (1.27) | (1.18) |
| *Living situation (ref: living with partner in the same household)* |  |  |
| Living with partner without a common household | 0.31 | 0.32 |
|  | (0.85) | (0.76) |
| Partner deceased or widowed | -4.02** | -4.01** |
|  | (1.52) | (1.45) |
| Single or divorced | 0.22 | 0.28 |
|  | (0.76) | (0.68) |
| Migration background (ref: no) | 0.22 | 0.15 |
|  | (0.88) | (0.81) |
| **Access factors** |  |  |
| Private health insurance (ref: statutory health insurance) | -0.16 | -0.26 |
|  | (0.67) | (0.61) |
| *Internet connection quality (ref: fast and stable)* |  |  |
| Fast, but not stable | -1.29 | -1.22 |
|  | (0.81) | (0.75) |
| Stable, but not fast | -2.85 | -2.82+ |
|  | (1.77) | (1.48) |
| Neither fast nor stable or no internet connection at home | 3.45+ | 3.36+ |
|  | (2.04) | (1.93) |
| **Health factors** |  |  |
| Number of psychiatric diagnoses | -0.35+ | -0.36+ |
|  | (0.21) | (0.19) |
| Presence of at least one chronic physical illness (ref: no) | 0.27 | 0.38 |
|  | (0.63) | (0.56) |
| Self-rated health | 0.54 | 0.61 |
|  | (0.48) | (0.43) |
| **Psychosocial factors** |  |  |
| Loneliness | -1.46* | -1.42* |
|  | (0.63) | (0.57) |
| Self-efficacy | 0.19 | 0.21 |
|  | (0.47) | (0.43) |
| **Personality** |  |  |
| Conscientiousness | 0.06 | 0.09 |
|  | (0.12) | (0.11) |
| Extraversion | -0.16 | -0.16 |
|  | (0.10) | (0.10) |
| Agreeableness | -0.22+ | -0.21* |
|  | (0.12) | (0.11) |
| Openness | 0.09 | 0.08 |
|  | (0.11) | (0.10) |
| Neuroticism | 0.10 | 0.11 |
|  | (0.10) | (0.09) |
| **COVID-19-related factors** |  |  |
| Received COVID-19 vaccination (ref: no) | -0.81 | -0.74 |
|  | (0.85) | (0.77) |
| Fear of COVID-19 | 0.15*** | 0.14*** |
|  | (0.04) | (0.04) |
| **Patient preferences** |  |  |
| Attitude towards telemental health services | 0.22*** | 0.22*** |
|  | (0.05) | (0.05) |
| Technology commitment | 0.00 | -0.01 |
|  | (0.05) | (0.05) |
| **Provider characteristics** |  |  |
| Provider attitude towards telemental health services | 1.33** | 1.43*** |
|  | (0.43) | (0.38) |
| Provider skills for using telemental health services | 0.62 | 0.66+ |
|  | (0.45) | (0.40) |
| Constant | 9.03* | 8.19* |
|  | (4.33) | (3.96) |
|  |  |  |
| **Observations** | 256 | 266 |
| **R-squared** | 0.51 | 0.51 |

*Notes.* Beta coefficients are reported. Robust standard errors in parentheses. ref = reference category. *** P<.001, ** P<.01, * P<.05, +P<.10.
